# Pharmaceuticals in source waters of 95 First Nations in Canada

**DOI:** 10.17269/s41997-021-00499-3

**Published:** 2021-06-28

**Authors:** Harold Schwartz, Lesya Marushka, Hing Man Chan, Malek Batal, Tonio Sadik, Amy Ing, Karen Fediuk, Constantine Tikhonov

**Affiliations:** 1 First Nations and Inuit Health Branch (FNIHB), Indigenous Services Canada, Ottawa, ON Canada; 2 Department of Biology, University of Ottawa, 30 Marie Curie, Ottawa, ON K1N 6N5 Canada; 3 Département de Nutrition, Faculté de Médecine, Université de Montréal, Pavillon Liliane de Stewart, C.P. 6128, succ. Centre-Ville, Montréal, QC, H3T 1A8 Canada; 4 Centre de recherche en santé publique de l’Université de Montréal et du CIUSS du Centre-sud-de-l’Île-de-Montréal (CReSP), 7101 avenue du Parc, Montréal, H3N 1X7 QC Canada; 5 Assembly of First Nations, 55 Metcalfe Street, Suite 1600, Ottawa, ON K1P 6L5 Canada

**Keywords:** Pharmaceuticals, First Nations, Surface water, Human health risk, Produits pharmaceutiques, Premières Nations, eaux de surface, risque pour la santé humaine

## Abstract

**Objectives:**

Pharmaceuticals are emerging contaminants in the environment. Little has been published about the presence of pharmaceuticals in waterbodies nearby or on reserve land of First Nations in Canada. The objectives of this study were to (1) quantify the level of pharmaceuticals in First Nations’ surface waters, (2) calculate the human health risks of the mixtures found, and (3) measure the exposure to pharmaceuticals in First Nations’ drinking water where source water was highly contaminated.

**Methods:**

This participatory study measured the levels of 43 pharmaceuticals from surface water samples taken at three water sampling sites chosen by the 95 participating First Nations. The sites were in proximity to recreational areas, fishing areas, drinking water sources, and/or wastewater outflows. When elevated levels of pharmaceutical mixtures were found in samples, drinking water samples were obtained and analyzed for potential pharmaceuticals. Human health risks were calculated by an established protocol.

**Results:**

In total, 432 samples were collected at 302 water sampling sites (285 surface water, 11 drinking water, and 6 wastewater sites). Quantifiable levels of 35 pharmaceuticals were found in 79 of the 95 (83%) participating First Nations at 193 of the 285 surface water sites (68%). Overall, the levels found were comparable to or lower than those found in other studies in Canada and worldwide.

**Conclusion:**

In almost all participating First Nations, there is no human health risk from consuming surface water for drinking. However, surface water in the vicinity of major urban centres should not be used as secondary untreated water sources due to the elevated human health risk associated with exposure to the mixtures of multiple pharmaceuticals detected.

**Supplementary Information:**

The online version contains supplementary material available at 10.17269/s41997-021-00499-3.

## Résumé

**Objectifs:**

Les produits pharmaceutiques sont des contaminants émergents dans l’environnement. Peu d’articles ont été publiés sur la présence de produits pharmaceutiques dans les plans d’eau à proximité ou sur les terres des réserves des Premières Nations au Canada. Les objectifs de cette étude étaient de 1) quantifier le niveau de produits pharmaceutiques dans les eaux de surface des Premières Nations, 2) calculer les risques pour la santé humaine des mélanges trouvés, et 3) mesurer l’exposition aux produits pharmaceutiques dans l’eau potable des Premières Nations où la source d’eau était fortement contaminée.

**Méthodes:**

Cette étude participative a mesuré les niveaux de 43 produits pharmaceutiques à partir d’échantillons d’eau de surface prélevés dans trois sites d’échantillonnage d’eau choisis par les 95 Premières Nations participantes. Les sites se trouvaient à proximité d’aires récréatives, de pêche, d’eau potable et/ou d’eaux usées. Lorsque des niveaux élevés de mélanges pharmaceutiques ont été trouvés dans les échantillons, des échantillons d’eau potable ont été prélevés et analysés pour la présence possible de produits pharmaceutiques. Les risques pour la santé humaine ont été calculés selon un protocole établi.

**Résultats:**

Au total, 432 échantillons ont été prélevés sur 302 sites d’échantillonnage d’eau (285 eaux de surface, 11 sites d’eau potable et 6 sites d’eaux usées). Des niveaux quantifiables de 35 produits pharmaceutiques ont été trouvés dans 79 des 95 (83 %) Premières Nations participantes, soit sans 193 des 285 sites d’eau de surface (68 %). Dans l’ensemble, les niveaux trouvés étaient comparables ou inférieurs à ceux trouvés dans d’autres études au Canada, aux États-Unis et dans le monde.

**Conclusion:**

Dans presque toutes les Premières Nations participantes, il n’y a aucun risque pour la santé humaine lié à la consommation d’eau de surface pour la boisson. Cependant, les eaux de surface à proximité des grands centres urbains ne devraient pas être utilisées comme sources d’eau secondaires non traitées en raison du risque élevé pour la santé humaine associé à l’exposition aux mélanges de plusieurs produits pharmaceutiques détectés.

## Introduction

Pharmaceuticals are synthetic or natural chemicals found in prescription medicines, over-the-counter and veterinary therapeutic drugs used for the diagnosis, cure, treatment, or prevention of diseases in humans and animals (WHO 2012). These include a wide range of antibiotics, analgesics, steroids, antidepressants, stimulants, antihypertensives, antidiabetics, and many other chemicals that are widely used for different purposes and are being discharged into the natural environment. Exposure to low doses of these emerging chemicals can impact non-target aquatic organisms and induce undesirable physiological effects on humans. Therefore, the presence of pharmaceuticals in the aquatic environment may pose health risks to humans and ecosystems (Sui et al. 2015).

Pharmaceutical chemicals may enter the environment through sewage discharge, livestock breeding, and landfill leachates. Other sources of pharmaceuticals include the direct disposal of unused drugs, the excretion of the compounds by humans and animals, and the effluents from drug manufacture (Kleywegt et al. 2019). The major pathway of pharmaceuticals release into the freshwater environment is through wastewater treatment plants (WWTPs) (Koné et al. 2013; Ebele et al. 2017). Since conventional wastewater treatment processes do not entirely eliminate pharmaceuticals, these compounds are frequently detected in surface water at low concentrations (Sui et al. 2015). Pharmaceuticals and their metabolites can also enter the waterways through run-off from land treated with sludge for agricultural purposes (Topp et al. 2008; Sabourin et al. 2009). Also, veterinary pharmaceuticals may contaminate the soil when animal wastes are sprayed on the agricultural field as fertilizers. Consequently, agricultural run-off can enter freshwater systems and leach into surface water and groundwater (Ebele et al. 2017; Sui et al. 2015). Depending on environmental conditions (temperature, pH) and physicochemical properties, some pharmaceuticals can be easily degraded, whereas others have the potential to persist in the aquatic environment for months to years (Sui et al. 2015). For instance, paracetamol (acetaminophen) and ibuprofen are rapidly degraded in water systems with DT50 (dissipation time of 50% of the amount originally present) ranging between 3.1 and 7 days whereas clofibric acid, diazepam, and carbamazepine are highly persistent with DT50 ranging from 119 to 328 days (Ebele et al. 2017).

The detrimental effects of pharmaceuticals on aquatic organisms have been well documented (Ortiz de Garcia et al. 2014; Ebele et al. 2017). The health risks depend on the nature and concentrations of the pharmaceuticals (Ebele et al. 2017). Some pharmaceuticals, such as sex hormones, glucocorticoids, and veterinary growth hormones, may interfere with the endocrine system and disrupt homeostasis in fish (WHO 2012). For example, exposure to 17α-ethynylestradiol was reported to cause feminization of male fish (Laurenson et al. 2014). Other pharmaceuticals, such as non-steroidal anti-inflammatory drugs (e.g., diclofenac, ibuprofen, and ketoprofen), have been linked to cardiac abnormalities, lowered heart rate in fish, and teratogenicity in fish embryos (Corcoran et al. 2010). Antibiotics present in the aquatic environment were reported to produce toxic effects on green algae, *Daphnia magna*, cyanobacteria, duckweed, crustaceans, and some fish species such as fathead minnow (Singer et al. 2016; Ebele et al. 2017). Furthermore, exposure to antibiotics may lead to a reduction in microbial biodiversity and the development of antibiotic-resistant bacteria (Singer et al. 2016; Grenni et al. 2018). A study in a First Nations in Manitoba investigated the prevalence of antibiotic resistance genes in source and drinking water (Fernando et al. 2016). The study detected various antibiotic resistance genes, namely, ampC (β-lactam resistance), tet(A) (tetracycline resistance), and mecA (methicillin resistance). In addition, five β-lactamase genes, responsible for resistance to β-lactam antibiotics (e.g., penicillin and cephalosporins), and six carbapenemase genes, responsible for resistance to carbapenems, were detected in both the source and drinking water samples (Fernando et al. 2016).

Over the past two decades, there has been considerable interest concerning the occurrence of pharmaceuticals in the environment. Many studies have been carried out in Canada (Kleywegt et al. 2011, 2019; Sultana et al. 2016; Waiser et al. 2011; Grill et al. 2016), the United States (Furlong et al. 2017; Glassmeyer et al. 2017), and globally (López-Pacheco et al. 2019) to quantify levels of pharmaceuticals in different environmental matrices. The concentrations of pharmaceuticals in the environment vary between countries and by regions of the same country and, generally, are correlated to the population density, the most frequently used classes of pharmaceuticals as well as the location of point sources, such as pharmaceutical production facilities (Ebele et al. 2017).

In Canada, studies have shown that municipal WWTPs are the major source of pharmaceuticals discharged into surface water which is used for drinking water production. Since WWTPs and drinking water treatment facilities are not designed to completely remove pharmaceuticals, these can be detected in treated drinking water (Koné et al. 2013). The Ontario Ministry of the Environment collected 258 samples from different source waters and drinking water systems and analyzed them for 48 pharmaceuticals and hormones. Overall, 27 chemical compounds were detected in source water and finished drinking water. Carbamazepine, gemfibrozil, and ibuprofen were the most frequently detected compounds in finished drinking water at concentrations 4 to 10 times lower than those found in the source water (Kleywegt et al. 2011). Metcalfe et al. (2014) monitored the concentrations of several pharmaceuticals (namely, carbamazepine, trimethoprim, sulfamethoxazole, ibuprofen, gemfibrozil, and estrone) in raw and treated drinking water in five drinking water treatment plants (DWTPs) in Ontario. Five pharmaceuticals were detected in treated drinking water with the average concentrations ranging from 0.04 to 4.29 ng/L of carbamazepine, 1.08 ng/L of trimethoprim, 0.08–0.19 ng/L of ibuprofen, 0.03–0.11 ng/L of estrone, and 0.02 ng/L of gemfibrozil (Metcalfe et al. 2014). Khan and Nicell (2015) summarized available data on pharmaceuticals in Canadian drinking waters. A total of 5813 samples of treated drinking water were analyzed to detect 47 unique pharmaceuticals. Among those, 20 pharmaceuticals were detected in 170 samples (2.9%). Ibuprofen and carbamazepine were the most commonly detected, with overall detection frequencies of 22% and 19%, respectively. Ethinylestradiol, indomethacin, chloramphenicol, doxycycline, and warfarin were not detected. The highest concentrations were reported for carbamazepine (601 ng/L) and erythromycin (155 ng/L) followed by ibuprofen (75 ng/L), roxithromycin (41 ng/L), acetaminophen (29 ng/L), and naproxen (26 ng/L). The maximum concentrations of tetracycline and trimethoprim were at 15 ng/L, ciprofloxacin—7 ng/L, gemfibrozil—4 ng/L, fluoxetine—6 ng/L, sulfamethoxazole—2 ng/L, clofibric acid—1.1 ng/L, and ketoprofen and estrone—1 ng/L (Khan and Nicell 2015). To date, there are no Canadian drinking water guidelines for pharmaceuticals; however, some other jurisdictions have developed varying guidelines to mitigate concerns about the consumption of recycled water that may contain quantifiable levels of pharmaceuticals. In 2008, Australia developed guidelines for pharmaceuticals for recycled water (Australian Guidelines 2008). In 2010, the state of California developed monitoring trigger levels for pharmaceuticals, which are essentially guidelines (Anderson et al. 2010). In 2011, New York City issued its standards for pharmaceuticals in drinking water (NYCEP 2011).

Many First Nations in Canada, especially those in remote and isolated regions, experience challenges to safe drinking water access (Patrick 2011; Bradford et al. 2016). Deficient water treatment facilities in First Nations, lack of trained operators, high operation and maintenance costs, and the relatively small population base of each First Nation represent main barriers to safe drinking water (Plummer et al. 2013). The Government of Canada and First Nations are working in partnership to improve the water infrastructure and support access to clean drinking water; nevertheless, some First Nations still have long-term drinking water advisories in place (ISC 2020). During the planning stage (2006–2007) of the participatory First Nations Food, Nutrition and Environment Study (FNFNES), data concerning the presence of pharmaceuticals in waterways within and nearby First Nations were nonexistent. First Nations sometimes use nearby water as a secondary water source in times of drinking water shortage. For example, during drinking water advisories, some First Nation members fill containers in local streams, rivers, and lakes to supplement their water supply. The presence of pharmaceuticals in water sources used for human consumption has raised concerns over potential human health risks from exposure to pharmaceuticals in drinking water. In fact, at low concentrations, pharmaceuticals may interfere with non-targeted receptors resulting in potentially harmful effects on non-target organs. For example, estrogenic pharmaceuticals may have adverse effects on hormonal control while antibiotics may contribute to antibacterial resistance (Singer et al. 2016; Houtman et al. 2014; Sui et al. 2015). Therefore, monitoring of pharmaceuticals in source water component was included in the study design of FNFNES. Previous risk assessments of individual pharmaceuticals have indicated that the levels found in this study are unlikely to pose human health risks (WHO 2012); however, the mixtures of pharmaceuticals at low concentrations may result in synergistic effects and significant ecotoxicity (Ebele et al. 2017; Houtman et al. 2014). Therefore, we characterized the human health risk of pharmaceuticals exposure from the use of surface waterbodies in First Nations for drinking.

The main goals of the pharmaceutical component of the FNFNES were to (1) establish a baseline of agricultural, veterinary, and human pharmaceuticals in the waters of First Nations in Canada; (2) determine the exposure of fish and shellfish (an important component of many First Nations’ diets) to pharmaceuticals in the surface waters in First Nations; (3) establish a priority list for future health and environmental effects studies (Chan et al. 2019); and (4) assess the human health risk of the pharmaceutical mixtures found in the participating First Nations.

## Methods

### Study design

The details of the study design and sampling of First Nation communities as well as the participatory process of this study are described in Chan et al. (2021) in this CJPH special issue. In total, 93^1^ First Nations completed all five components of FNFNES, and 95 First Nations participated in the pharmaceutical component. In Manitoba, 3 out of 12 First Nations collaborated on completing pharmaceutical sampling despite withdrawing from other components of the study. One Ontario First Nation withdrew from the pharmaceutical component of the study. Thus, 95 First Nations participated in the pharmaceutical component of the FNFNES (93 +3 – 1 = 95).

**Fig. 1:**
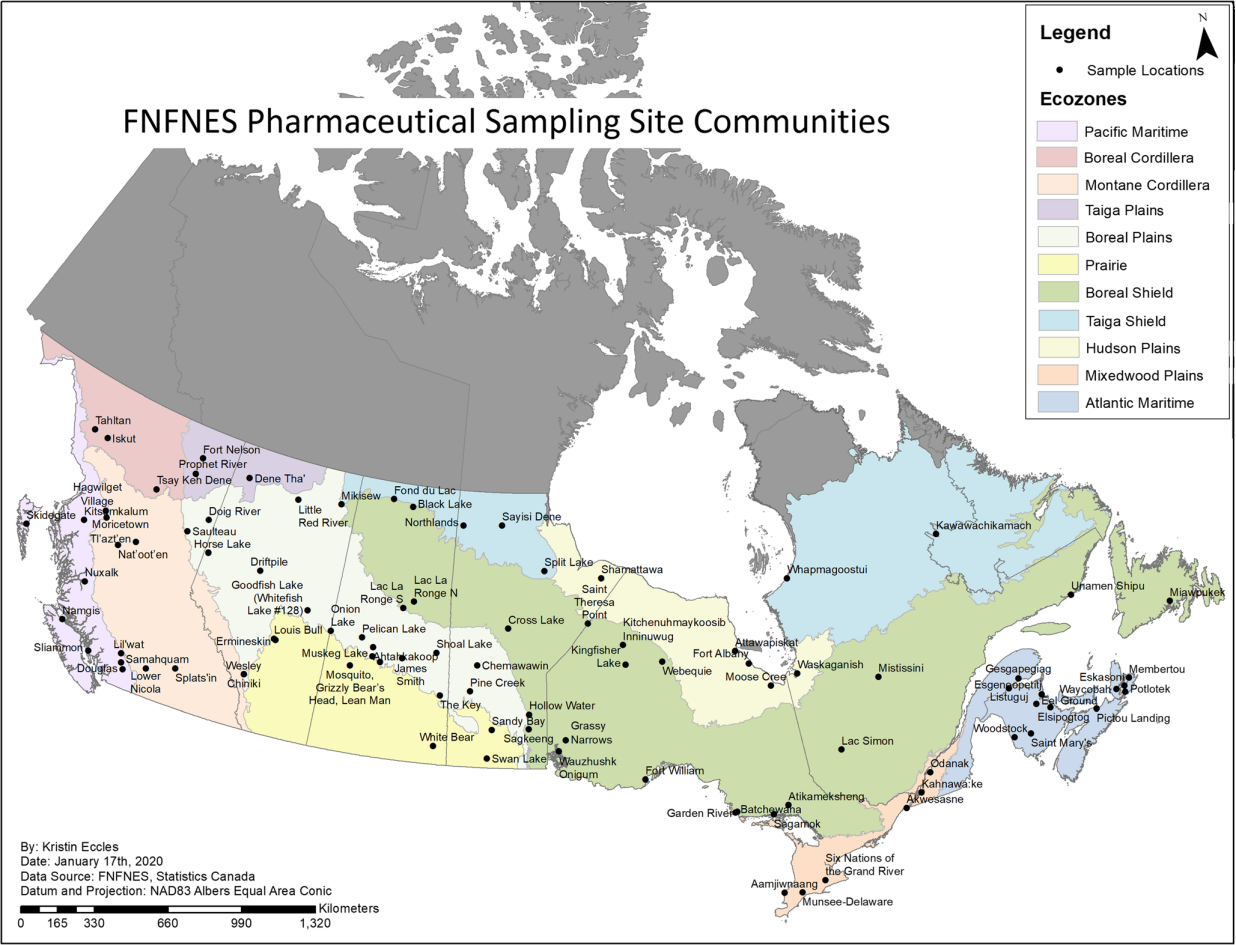
First Nations participating in the pharmaceutical sampling of the FNFNES

Eleven ecozones were identified based on ecosystems (Wiken EB 1986) whereas the defined regions largely reflected provincial boundaries, except for Labrador which was included in the Quebec region and the Atlantic region which included the three Maritime provinces and Newfoundland. An ecozone is a large geographical region characterized by distinct biodiversity of plants and animals along with geographical characteristics and climate (Wiken EB 1986, ecozones.ca). The ecozones are described in Chan et al. (2021) and presented in Fig. 1. In this study, the results on pharmaceuticals in source water are presented by region and ecozone levels.

### Pharmaceutical sampling sites

First Nations leadership (Chief and Band Council) chose the sampling sites. In British Columbia and Quebec, the most popular sites for sampling were recreational sites, such as swimming areas. In Manitoba and Ontario, sites near the drinking water intakes were the most often chosen. In Alberta, Saskatchewan, and Atlantic Canada, fishing sites were most popular for the sampling. The water samples were usually collected by the First Nations and Inuit Health Branch’s (FNIHB) Environmental Public Health Officers (EPHOs) or the First Nations’ EPHOs in collaboration with local First Nation representatives. Where sampling required the use of a boat, at least two individuals participated in the sampling. One First Nation requested sampling at a remote location not accessible to the community via ATV; therefore, a flight was chartered to complete the sampling (Table 2).

Each participating First Nation determined the location of three surface water sites. The most prevalent sites chosen were (1) swimming areas and/or proximity to the community (30%), (2) fishing sites (29%), (3) near the sources of the community’s drinking water (25%), and (4) proximity to wastewater (18%). Also, two Ontario First Nations, where elevated levels of multiple pharmaceuticals were detected in surface water, requested a sampling of drinking water: one where the drinking water source was surface water and one where the source was groundwater. In addition, two drinking water sites in one First Nation, where the source water was surface water, were sampled upon request in Quebec. In total, groundwater was sampled in two communities, one in Ontario and one in Alberta (five sites in one First Nation in Ontario and one site in Alberta). In addition, five First Nations in Ontario, Alberta, and Saskatchewan requested that sampling take place in their wastewater sites. One Saskatchewan First Nation also requested sampling in a pond situated in the middle of their garbage dump. Since ducks and other birds were often seen in the community lagoons and wastewater, there was a concern that the wastewater may be a potential route of exposure to contaminants to the First Nation members who consume these birds. Therefore, in total, 6 wastewater sites were sampled (5 lagoons and one dump site).

### Choice of pharmaceuticals

The criteria used for the selection of pharmaceuticals were (1) levels of detection of the pharmaceuticals in the aquatic environment in previous studies; (2) frequency of detection of the pharmaceuticals in the environment in previous studies; and (3) evidence of usage of the pharmaceuticals in the vicinity of a First Nation. Information on the First Nations’ pharmaceutical usage was shared by the Non-Insured Health Benefits Program of the FNIHB of Indigenous Services Canada (Chan et al. 2019). Forty-three pharmaceuticals were included based on their use in human medicine, veterinary drugs, and aquaculture. The selected pharmaceuticals, which included analgesics, antacids, antibiotics, anticoagulants, antidepressants, antidiabetics, antihistamines, antihypertensives, diuretics, lipid regulators, steroids, and synthetic contraceptives, have been found to persist in the environment (Ebele et al. 2017). Illicit drugs were not measured.

### Laboratory analyses

Cantest/Maxxam in Vancouver, British Columbia (2009 to 2011) and ALS Global in Waterloo, Ontario (2012 to 2016) were awarded contracts to analyze samples based on rigorous performance evaluations and a formal bidding process. A comprehensive quality assurance/quality control (QA/QC) program was executed by the analytical laboratories and the QA/QC results were verified and approved by the Principal Investigators of the FNFNES.

#### QA/QC program

For a batch of 20 samples, there was a method blank (MB) where the measured level was less than the limit of reporting (LOR); a laboratory control sample (LCS) where the sample is spiked with the target analytes and recovery was within the control limits of 50 to 150%; a matrix spike (MS) where recovery of a known amount of target analytes was 60 to 140%; a duplicate sample where the acceptance criteria were +/- 40%.

#### Matrix effects

Matrix effects were corrected via dilution when needed.

#### Instrumentation

The pharmaceutical analysis was carried out on a Sciex 5500 Q-TRAP LC/MS/MS. There were eight analytical methods used for the quantitation of the 43 pharmaceuticals measured in the study. The HPLC gradients and columns used are listed in the instrumentation conditions table (see Supplemental material: Analytical Methodology).

#### Isotopically labelled standards

The eight isotopically labelled standard (ISTD) mixtures that were used for each of the methods are provided in the instrumentation conditions table (Supplemental material: Analytical Methodology).

The limits of quantitation (LOQ) were based on previously established minimum reporting levels of the University of Northern British Columbia (UNBC). The limits of detection (LOD) were determined by a signal-to-noise (S/N) ratio of 3:1. The LOD and LOQ for the analytes are provided in the Instrument Conditions table (see Supplemental material: Analytical Methodology).

### Sample preparation 2009 to 2011

Two separate 250 mL sample aliquots were required to analyze all of the target analytes (*n* = 43).

One aliquot was adjusted to pH 1.95–2.0 and mixed with 500 mg of Na4EDTA·2H2O. The sample was loaded onto a HLB solid-phase extracting column (supplier, Oasis). The column is washed with 10 mL water and eluted with 12 mL of methanol. The eluent was evaporated and reconstituted with 450 μL water and 50 μL internal standard (ISTD) (See nine pharmaceutical methods for the composition of the ISTD (see Supplemental material: Analytical Methodology)). Using a nitrogen evaporator, the elutant was evaporated to near dryness then topped up to 450 uL with HPLC water (isotopically labelled standards were then added). The extract was analyzed by LCMSMS (SCIEX Q-Trap 5500) in positive and negative modes.

### Metformin

The second 250 mL aliquot was adjusted to pH 10 ± 0.5. The sample was loaded onto a HLB solid-phase extracting column (Oasis). The column was eluted with 6 mL of methanol followed by 9 mL of 2% formic acid in methanol. The eluent was evaporated and reconstituted with 450 μL acetonitrile and 50 μL internal standard (See the pharmaceutical method—Using a nitrogen evaporator, the elutant was evaporated to near dryness then topped up to 450 uL with HPLC water, isotopically labelled standards were then added (see Supplemental material: Analytical Methodology)). The extract was analyzed by LCMSMS in positive ion mode.

### 17α-ethinylestradiol

A 20 mL aliquot of the sample was loaded onto an HLB SPE column (Oasis). The column was washed with 3 mL of water and eluted with 3 mL of methanol. The eluent was evaporated to dryness. One hundred microlitres of 100 mM sodium bicarbonate (pH 10.5) was added followed by 100 μL of 1 mg/mL dansyl chloride to derivatize the ethinylestradiol. Samples were then incubated at 60°C for 6 min. After cooling to room temperature, the samples were diluted with 50 μL of 1:1 acetonitrile:water. The extracts were analyzed by LCMSMS in positive ion mode.

### Sample preparation 2012–2016

From 2012 to 2016, an aliquot of the water sample is filtered with 0.2 μm RC membrane syringe filters. HPLC methanol and internal standards are added (10 mL of sample and 1 mL of solvent). The water sample is then vialed and analyzed by LCMSMS with direct injection without sample cleanup (PPCP groups 1–3, caffeine method, tetracycline method, and negative ion mode (PPCP - Neg).

A second aliquot of the water sample is filtered with 0.2 μm RC membrane syringe filters. HPLC acetonitrile and internal standards are added (10 mL of sample and 0.5 mL of solvent). The water sample is then vialed and analyzed by LCMSMS with direct injection without sample cleanup in positive ion mode (metformin method).

For 17α-ethinylestradiol and the trenbolones, a 100 mL aliquot of the sample was spiked with the ISTD.

The sample was loaded onto a HLB SPE column (Oasis) which was preconditioned with HPLC MeOH followed by HPLC water. The column was dried and eluted with 10 mL of 0.1% formic acid in HPLC MeOH and 5mL of MeOH/acetone 1:1. The elutant was evaporated on an N-Evap to near dryness and the volume was adjusted to 1 mL with 10% MeOH in water. The initial volume was 100 mL—final volume 1.0 mL (steroid and hormone method).

### Human health risk assessment of the mixture of pharmaceuticals

Risk assessment exposure to pharmaceuticals in drinking water is usually assessed separately for each pharmaceutical using the minimum therapeutic dose (MTD) or the lowest dose of a drug that produces a desired therapeutic effect in a target population, as a point of departure (PoD) (Houtman et al. 2014; Khan and Nicell 2015; WHO 2012). Similar to the lowest-observed-adverse-effect level (LOAEL), the MTD represents the lowest concentration at which undesirable adverse or toxic effects on human health can be observed (WHO 2012) and is used when toxicological data to derive the no-observed-adverse-effect level (NOAEL) or the LOAEL or a benchmark dose are not available. A screening reference value (an acceptable daily intake (ADI)) is estimated by dividing the MTD by uncertainty factors (UFs) to reflect uncertainties in extrapolation from experimental animals to humans and the variation within the exposed population (Houtman et al. 2014; Khan and Nicell 2015; WHO 2012).

The human health risk from a mixture of pharmaceuticals found in surface water in First Nations was assessed using a method first proposed by Houtman et al. (2014). As per this method, the risk to human health is calculated based on lifelong exposure (with an average life expectancy of 70 years). For each pharmaceutical, a provisional guideline value (pGLV) (i.e., a safe dose below which no adverse effects will occur) is calculated based on the ADI and standard body weight (bw) (70 kg for men and 60 kg for women) along with the standard assumption that an adult is consuming 2 L of water a day. The authors assume that only 10% of the total exposure comes from the ingestion of drinking water as there are several sources of pharmaceutical exposure.

This is shown in Eq. 1:

1 $$ pGLV= ADI\ \left(\mu g/ kgbw/ day\right)\times bw\ (kg)\times 0.1/2\ L $$

Then, the benchmark quotient (BQ) is calculated from the maximum concentration of each pharmaceutical at that site divided by the pGLV as shown in Eq. 2:

2 $$ BQ= maximum\ FNFNES\ concentration\  at\  the\ site\ \left(\mu g/L\right)/ pGLV $$

Finally, to estimate the possible health risk of the total mixture of pharmaceuticals present in water, the risk quotient (RQ) is calculated as the sum of the BQs as seen in Eq. 3:

3 $$ RQ=\sum BQs $$

An RQ value greater than one is considered to represent a human health risk when drinking the water over a period of 70 years.

Human health risk assessment was completed for potential exposure to multiple pharmaceuticals for all samples. The results for four of the participating First Nations with the greatest number of pharmaceuticals in their waters are as follows:

1. One First Nations community located in the Boreal Shield ecozone in Ontario with 18 unique pharmaceuticals detected;
2. One First Nations community located in the Mixedwood Plains ecozone in Ontario with 17 unique pharmaceuticals found, including 17α-ethinylestradiol (a commonly used contraceptive);
3. One First Nations community in the Mixedwood Plains ecozone in Ontario with 18 different pharmaceuticals, but with no contraceptive pharmaceutical detected; and
4. One Prairies wastewater site with higher concentrations of pharmaceuticals compared with those found in the surface water sites.

## Results and discussion

Overall, 35 out of 43 unique pharmaceuticals were detected at one or more surface water sites (Table 1). Pharmaceuticals were detected in 79 of the 95 (83%) First Nations participating in the pharmaceutical component of the study (Fig. 1). In total, 432 samples were collected at 302 water sampling sites (Table 2). Of those, 285 were surface water sites, 11 were drinking water sites (in four communities), and 6 were wastewater sites (in five communities). Pharmaceuticals were found in 193 of the 285 surface water sites (68%), in 7 of the 11 drinking water sites, and in all (6/6) wastewater sites. At drinking water sites, 3 unique pharmaceuticals were found in two First Nations where the source was surface water, and 2 pharmaceuticals were detected in groundwater sites. At the wastewater sites, 28 distinct pharmaceuticals were detected.

**Table 1:** Pharmaceuticals analyzed and detected in the FNFNES from 2009 to 2016

Pharmaceutical	Detected
Analgesic	
Codeine	x
Analgesic/anti-inflammatory	
Acetaminophen	x
Diclofenac	x
Ibuprofen	x
Indomethacin	
Ketoprofen	x
Naproxen	x
Antacid	
Cimetidine	x
Ranitidine	x
Antibiotic	
Chlortetracycline	x
Ciprofloxacin	x
Clarithromycin	x
Erythromycin	x
Isochlortetracycline	x
Lincomycin	
Monensin	
Oxytetracycline	
Roxithromycin	
Sulfamethazine	x
Sulfamethoxazole	x
Tetracycline	
Trimethoprim	x
Antianginal	
Dehydronifedipine	x
Antidiabetic	
Metformin	x
Pentoxifylline	x
Anticoagulant	
Warfarin	x
Anticonvulsant	
Carbamazepine	x
Antidepressant	
Fluoxetine	x
Antihistamine	
Diphenhydramine	x
Antihypertensive (beta-blocker)	
Atenolol	x
Metoprolol	x
Antihypertensive	
Diltiazem	x
Diuretic	
Furosemide	x
Hydrochlorothiazide	x
Lipid regulators	
Bezafibrate	x
Clofibric acid	x
Gemfibrozil	x
Nicotine metabolite	
Cotinine	x
Oral contraceptive	
17α-Ethinylestradiol	x
Statin	
Atorvastatin	x
Steroid	
17α-Trenbolone	
17β-Trenbolone	
Stimulant	
Caffeine	x

**Table 2:** Summary of the FNFNES pharmaceutical sampling sites of First Nations

Year	Region	No. of First Nations*	Recreational and/or proximity to a community	Fishing sites	Drinking water sources	Proximity to wastewater	Total no. of sites
2009	British Columbia	21	29	10	10	12	61
2010	Manitoba	12	11	9	12	4	36
2011/2012	Ontario	17	16	9	30	11	66
2013	Alberta	10	7	11	9	3	30
2014	Atlantic Canada	11	4	21	2	7	34
2015	Saskatchewan	14	12	18	2	10	42
2016	Quebec	10	10	8	9	6	33
Total		95	89	86	74	53	302

*While 95 First Nations completed the pharmaceutical component of the study, 3 First Nations in Manitoba withdrew from the larger study but continued to collaborate on the pharmaceutical study. One Ontario First Nation community withdrew from the pharmaceutical sampling component while participating in all other components of the FNFNES study in Ontario

When pharmaceuticals were detected, attempts were made to trace the source by comparing them to the lists of pharmaceuticals prescribed in that community. However, visitors to the community who could have brought pharmaceuticals from outside the community (especially with very long half-lives such as clofibric acid) made it difficult to account for the true source of the human pharmaceuticals detected (Chan et al. 2019). The veterinary antibiotic sulfamethazine was traced back to the use on dogs in one Ontario community.

### Pharmaceuticals in surface water

Table 3 summarizes data (e.g., mean, median, min, max, frequency of detection, and detection limits) on pharmaceuticals concentrations in surface water in First Nations communities. The occurrence of pharmaceuticals by ecozone, the frequency of detection by communities, and maximum concentration are shown in Table 4 where pharmaceuticals are listed in order of prevalence at sampling sites. For example, caffeine (a component of the analgesic, acetaminophen/caffeine/codeine) was the most prevalent pharmaceutical detected and was found at 105 sites (36.8%), in 57 communities (61%), and in all 11 ecozones. Caffeine is also present in coffee, tea, cola drinks, and chocolate. Atenolol, a hypertensive, was found in 78 sampling sites (27.4%), in 28 communities and across 8 of the 11 ecozones. Atenolol is an antihypertensive medication that was among the topmost prescribed pharmaceuticals in First Nations where it was detected (Chan et al. 2019).

**Table 3:** Levels of pharmaceuticals found in surface water of the FNFNES

Pharmaceutical	*n*	Mean (ng/L)	Median (ng/L)	Min (ng/L)	Max (ng/L)	Frequency of detection (%)	Quantification limit (ng/L)
Acetaminophen	25	36.6	18.0	11.0	307	6.6	10
Atenolol	116	22.6	15.5	5.0	245	28.4	5.0
Atorvastatin	2	8.5	8.5	8.2	8.8	0.5	5.0
Bezafibrate	26	3.7	2.75	1.1	11.2	6.4	1.0
Caffeine	133	120.9	19.4	5.1	4018	32.5	5.0
Carbamazepine	57	13.3	8.1	0.6	39.6	14.0	0.5
Chlortetracycline	3	12.0	12.0	12.0	12.0	0.7	10
Cimetidine	57	6.4	3.3	2.1	40.9	14.0	2.0
Ciprofloxacin	15	28.2	29.0	20.0	37.7	3.7	20.0
Clarithromycin	33	15.6	9.1	2.1	69.6	8.1	2.0
Clofibric acid	10	3.2	2.7	1.3	8.6	2.5	1.0
Codeine	24	44.0	39.5	9.6	101	5.9	5.0
Cotinine	71	16.8	8.5	5.0	90.0	17.4	5.0
Dehydronifedipine	5	4.9	3.3	2.4	9.5	1.2	2.0
Diclofenac	14	22.9	21.0	15.0	38.0	3.4	15.0
Diltiazem	3	46	59.7	5.2	73.1	0.7	5.0
Diphenhydramine	9	24.8	14.0	12.0	56.0	2.2	10.0
Erythromycin	1	23.0	23.0	23.0	23.0	0.2	10.0
Fluoxetine	6	31.4	30.8	15.8	50.7	1.5	5.0
Furosemide	5	15.2	8.5	6.4	30.7	1.2	5.0
Gemfibrozil	15	4.4	2.5	1.1	16.8	3.7	1.0
Hydrochlorothiazide	26	43.9	49.9	5.1	85.9	6.4	5.0
Ibuprofen	13	117.2	85.0	27.0	367	3.2	20.0
Isochlortetracycline	1	13.0	13.0	13.0	13.0	0.2	10.0
Ketoprofen	19	26.0	6.4	2.4	307	4.7	2.0
Metformin	88	716	197.5	13.3	6210	21.6	10.0
Metoprolol	25	21.3	20.2	6.8	77.0	6.1	5.0
Naproxen	28	43.7	39.2	5.5	120	6.9	5.0
Pentoxifylline	6	13.7	11.1	2.5	26.9	1.5	2.0
Ranitidine	19	22.1	24.0	10.0	33.0	4.7	10.0
Sulfamethazine	19	15.4	16.4	6.1	24.2	4.7	5.0
Sulfamethoxazole	57	20.5	10.1	2.0	87.0	14.0	2.0
Trimethoprim	28	8.3	5.6	2.2	32	6.9	2.0
Warfarin	12	2.4	1.5	0.5	6.9	2.9	0.5
17α-Ethinylestradiol	6	0.54	0.50	0.38	0.74	1.5	0.2

Pharmaceuticals were detected in 408 water samples

**Table 4:** Pharmaceuticals detected in surface water of First Nations by ecozone

Pharmaceutical	FNFNES max ng/L	Quantification limit ng/L	No. of First Nations found	No. of sites found	% of total no. of sites*	Ecozone**
Caffeine	4018	5	57	105	36.8	1,2,3,4,5,6,7,8,9,10,11
Atenolol	245	5	28	78	27.4	1,3,5,6,7,9,10,11
Metformin	6210	10	27	60	21.0	5,6,7,8,9,10,11
Cotinine	90	5	28	50	17.5	3,5,6,7,8,9,10,11
Sulfamethoxazole	87	2	15	41	14.4	5,7,9,10,11
Carbamazepine	91.5	0.5	18	40	14.0	5,6,7,8,9,10,11
Cimetidine	40.9	2	15	37	13.0	4,5,6,7,8.10
Naproxen	244	5	13	24	8.4	6,7.9,10,11
Acetaminophen	307	10	13	23	8.1	1,3,5,6,7,8,9,10,11
Clarithromycin	69.6	2	10	23	8.1	4,5,7,10,11
Trimethoprim	32.0	2	9	20	7.0	1,2,5,7,9,10
Bezafibrate	11.2	1	8	19	6.7	5,7,10,11
Metoprolol	77.0	5	6	18	6.3	5,7,10,11
Ketoprofen	307	2	10	17	6.0	1,3,5,6,7,10,11
Codeine	101	5	6	16	5.6	7,9,10,11
Hydrochlorothiazide	85.9	5	6	16	5.6	7,9,10,11
Gemfibrozil	16.8	1	7	15	5.3	5,7,9,10
Ranitidine	33.0	10	4	12	4.2	9,10,11
Warfarin	6.9	0.5	5	11	3.9	1,3,7,10
Diclofenac	38	15	6	10	3.5	6,7,10,11
Clofibric acid	8.6	1	5	9	3.1	1,2,3,6
Ciprofloxacin	37.7	20	4	8	2.8	1,10
Sulfamethazine	24.2	5	4	8	2.8	10,11
Ibuprofen	367	20	5	7	2.5	7,9,10,11
Diphenhydramine	9.5	2	4	6	2.1	7,9,10,11
Fluoxetine	50.7	5	4	5	1.8	1,2,3,5
Dehydronifedipine	9.5	2	5	5	1.8	1,3,5,7
Pentoxifylline	26.9	2	3	5	1.8	1,7,11
17α-Ethinylestradiol	0.74	0.2	3	5	1.8	7,9,10
Furosemide	30.7	5	2	4	1.4	10,11
Chlortetracycline	12.0	10	2	3	1.1	5
Diltiazem	73.1	5	2	2	0.7	7,10
Atorvastatin	8.8	5	1	1	0.4	11
Erythromycin	23.0	10	1	1	0.4	7
Isochlortetracycline	13.0	10	1	1	0.4	8

*Total number of sites sampled: *n* = 285

** Ecozones: 1, Pacific Maritime; 2, Boreal Cordillera; 3, Montane Cordillera; 4, Taiga Plains; 5, Boreal Plains; 6, Prairies; 7, Boreal Shield; 8, Taiga Shield; 9, Hudson Plains; 10, Mixedwood Plains; 11, Atlantic Maritime

Cotinine, a nicotine metabolite, was found in 50 sites (17.5%), 28 First Nations, and eight ecozones. An average of 80% of nicotine that is consumed by people is excreted as cotinine. Its presence in surface water likely reflects contamination of urine of tobacco users (Chan et al. 2019).

Metformin, an anti-diabetic medication, was found in 60 sites (21%), 27 First Nations, and seven ecozones. Metformin is one of the most commonly prescribed medications to treat type 2 diabetes in the communities where it was detected. Heavy usage of this medication reflects the high rates of type 2 diabetes among First Nations (Batal et al. 2021). Metformin is not metabolized by humans and therefore, is discharged into sewage unchanged. It can be bacterially transformed (mainly in WWTPs) to the ultimate transformation product guanylurea (Trautwein et al. 2014; Godoy et al. 2018). Metformin and guanylurea present in the aquatic environment may cause behaviour changes and disturb the reproductive capabilities of fish living near sewage treatment outlets (MacLaren et al. 2018; Godoy et al. 2018).

The oral contraceptive, 17α-ethinylestradiol, was found in five sites (1.8%) in three communities in three ecozones: Boreal Shield (in Manitoba), Hudson Plains, and Mixedwood Plains (in Ontario). This pharmaceutical enters the water environment primarily through human excretion and the disposal of unused medications into toilets and sinks. Since municipal sewage treatment plants do not completely remove estrogens in the effluents, these compounds are directly discharged to the natural environment (Servos et al. 2005). Environmental estrogens, such as 17α-ethinylestradiol, can persist in water systems for several months and accumulate in fish and plants. Exposure to relatively low concentrations of 17α-ethinylestradiol has been shown to induce reproductive dysfunctions in fish, such as feminization in male fish, the formation of a female reproductive duct in the testis, induction of intersex, and reduced fertility (Laurenson et al. 2014).

Among all antibiotics tested, the most frequently detected were sulfamethoxazole (41 sites), clarithromycin (23 sites), and trimethoprim (20 sites) followed by ciprofloxacin (8 sites) and sulfamethazine (8 sites). These antibiotics were prescribed to treat different bacterial infections (skin, ear, bladder, kidney, and respiratory tract) in the communities where they were detected (Chan et al. 2019). Sulfamethazine was used to treat infection in dogs. Antibiotics in surface water were also detected in previous Canadian studies (Metcalfe et al. 2014; Khan and Nicell, 2015). Exposure to low concentrations of antibiotics may affect genetic and phenotypic variability and lead to the development of antibiotic-resistant bacteria (Grenni et al. 2018). This represents risks to both humans and wildlife. Chronic exposure to antibiotics was reported to cause autoimmune problems and an increase in infections in aquatic organisms (Singer et al. 2016). In humans, antibiotic resistance may lead to enhanced pathogenicity, disease outbreaks, and transmission resulting in prolonged morbidity and hospitalization (Singer et al. 2016; Grenni et al. 2018).

Table 5 presents the maximum concentrations of 35 unique pharmaceuticals detected in each ecozone. Most of the pharmaceuticals were present in low concentrations within an order of magnitude of the detection limit. A greater number of pharmaceuticals at higher concentrations were found in the more southern and eastern ecozones as seen in Table 4. Elevated levels of caffeine and metformin detected in surface water in four ecozones (Boreal Shield (Ontario Region), Hudson Plains Shield (Ontario Region), Mixedwood Plains Shield (Ontario Region), and Atlantic Maritime Shield (Quebec Region)) were found mainly due to the influence of wastewater. More detailed information on pharmaceuticals in the source water by ecozone can be found elsewhere (Chan et al. 2019).

**Table 5:** Maximum concentration (ng/L) of pharmaceuticals in surface water by ecozone

QL	Pharmaceutical	Pacific Maritime	Boreal Cordillera	Montane Cordillera	Taiga Plains	Boreal Plains	Prairies	Boreal Shield	Taiga Shield	Hudson Plains	Mixedwood Plains	Atlantic Maritime
5	Acetaminophen	17.5		13.8		17	64	307	24	25	20	124
5	Atenolol	6.7		5		28.7	17.9	245		105	42	24.3
5	Atorvastatin											8.8
1	Bezafibrate					2.9		11.2			7.8	1.1
5	Caffeine	19.4	51.9	9.2	8.4	160	30.5	355	40.1	4018	502	851
0.5	Carbamazepine					17.3	0.75	39.6	1.8	8.1	32.9	37.6
10	Chlortetracycline					12						
2	Cimetidine				3.3	5.6	40.9	2.9	5.1		4	
20	Ciprofloxacin	37.7									36	
2	Clarithromycin				9.4	4.1		69.6			35.3	21.3
1	Clofibric acid	4.1	8.6	2.3			4.4					
5	Codeine					14.7		101		62.5	101	9.6
5	Cotinine			6.6		8.5	16.7	46.2	56.6	43.8	31.3	90
2	Dehydronifedipine	9.5		3.3		3.1						
15	Diclofenac						35	2.4			38	16
5	Diltiazem							73.1			5.2	
10	Diphenhydramine							56		12	14	30
10	Erythromycin							23				
5	Fluoxetine	41.7	50.7	18.3		32.4						
5	Furosemide										12.5	30.7
1	Gemfibrozil					1.5		16.8		7.1	5.6	
5	Hydrochlorothiazide							5.6		37.9	85.9	38.7
20	Ibuprofen							53		367	85	150
10	Isochlortetracycline				13							
2	Ketoprofen	307		45.2		4.6	7.3	9.3			3.1	7.2
10	Metformin					93	41	5640	60	6210	2020	5880
5	Metoprolol					7		77			25.6	25.3
5	Naproxen						16.3	75		67.6	120	244
2	Pentoxifylline	4.5						12.7				26.9
10	Ranitidine									15	33	12
5	Sulfamethazine										19.1	24.2
2	Sulfamethoxazole					19		87		9.3	45.7	22
2	Trimethoprim	2.4	4.3			4.3		32		3.9	10.2	
0.5	Warfarin	6.9		3.9				2.9			0.51	
0.2	17α-Ethinylestradiol							0.45		0.55	0.74	

*QL*, quantitation limit

### Comparison of the levels of pharmaceuticals in surface water found in the FNFNES with those in other Canadian, American, and global studies

In Table 6, the maximum levels of the most prevalent pharmaceuticals found in the FNFNES are compared with the maximum values found in other Canadian (by province), American (by state), and global studies (by country). In general, pharmaceutical levels in the FNFNES were much lower than their levels reported across Canada, the USA, and worldwide. For example, the maximum level of 17α-ethinylestradiol found in the FNFNES was 0.74 ng/L. For comparison, in Quebec, Environment Canada reported a maximum concentration of 3.1 ng/L (Environment Canada 2012), which is over four times higher than the FNFNES level. In the USA, the US Environmental Protection Agency researchers found 431 ng/L of 17α-ethinylestradiol (Bai et al. 2018), which is more than 580 times the FNFNES maximum. In Brazil, researchers with the University of Brasilia reported a 17α-ethinylestradiol concentration of 5900 ng/L, which is over 7900 times the FNFNES value (Sodré et al. 2018). For a more detailed comparison of all 35 pharmaceuticals found in the FNFNES surface water, see Chan et al. (2019).

**Table 6:** Comparison of pharmaceutical levels detected in surface water in First Nations participating in the FNFNES with findings from other Canadian, American, and global studies*

Pharmaceutical detected	FNFNES max (ng/L)	Canada max (ng/L)	USA max (ng/L)	Global max (ng/L)
Analgesic/anti-inflammatory				
Acetaminophen	307	3500a, SK	10,000^b^	106,970^c^, Kenya
Antacid				
Cimetidine	40.9	5.33^g^, ON	688^h^, IA	1338^i^, Korea
Antibiotic				
Sulfamethoxazole	87.0	600a, SK	3280^h^, IA	252,082^j^, Vietnam
Anticonvulsant				
Carbamazepine	91.5	749^k^, ON	3480^l^, NJ	276,000^m^, Hungary
Antidiabetic				
Metformin	6210	10,100^g^, ON	34,000^n^, OH	20,015^o^, China
Antihypertensive (beta-blocker)				
Atenolol	245	204^g^, ON	1,850^p^, CO	39,100^q^ South Africa
Nicotine metabolite				
Cotinine	90	189^r^, AB	1400^s^, AZ	6582^t^, Spain
Oral contraceptive				
17α-Ethinylestradiol	0.74	3.1^w^, QC	431^p^, CO	5900^x^, Brazil
Stimulant				
Caffeine	4018	1960^g^, ON	7110^u^, MD	1,121,446^v^, Costa Rica

*Only the most prevalent pharmaceuticals found in the FNFNES are presented here. For a more detailed comparison of all 35 pharmaceuticals found in the FNFNES, see Chan et al. (2019)

^a^Waiser et al. (2011); ^b^Kolpin et al. (2002); ^c^K’oreje et al. (2016); ^d^Brun et al. (2006); ^e^Benotti and Brownawell (2009); ^f^Gumbi et al. (2017); ^g^de Solla et al. (2016); ^h^Bradley et al. (2014); ^i^Choi et al. (2018); ^j^Thai et al. (2018); ^k^Kleywegt et al. (2011); ^l^Roden (2013); ^m^Bókony et al. (2018); ^n^Elliott et al. (2018); ^o^Kong et al. (2015); ^p^Bai et al. (2018); ^q^Agunbiade and Moodley (2014); ^r^Sosiak and Hebben (2005); ^s^Chiu and Westerhoff (2010); ^t^Valcárcel et al. (2011); ^u^Young et al. (2008); ^v^Spongberg et al. (2011); ^w^Environment Canada (2012); ^x^Sodré et al. (2018)

### Comparison with ambient guidelines

In Canada, only one pharmaceutical, 17α-ethinylestradiol, has an ambient water guideline level at 0.5 ng/L in the province of British Columbia (Nagpal and Meays 2009). This pharmaceutical was found in samples collected from three First Nations, with concentration levels at 0.55 and 0.74 ng/L in two First Nations located in the Hudson Plains and Mixedwood Plains ecozones in Ontario and at 0.45 ng/L in one First Nation in Manitoba located in the Boreal Shield ecozone. The levels found in the two First Nations in Ontario were above the 30-day average concentration guideline level set by the province of British Columbia to protect aquatic life (0.5 ng/L) and therefore could affect the fertility of some fish (Laurenson et al. 2014). The levels of 17α-ethinylestradiol were below the maximum allowable guideline for a single value of 0.75 ng/L (Nagpal and Meays 2009).

### Pharmaceuticals in drinking water—post-study testing

Following communication of the initial pharmaceutical results, four First Nations (two located in Ontario, one in Quebec, and one in Alberta) requested that samples from the community drinking water system be tested for potential pharmaceutical contamination.

The first First Nation, located in the Mixedwood Plains ecozone in the Ontario region, had a community water treatment plant that delivered water to homes via a piped distribution system. Two pharmaceuticals, atenolol (an antihypertensive) and carbamazepine (an anticonvulsant), were detected in drinking water samples collected at the tap in three homes: the concentration of atenolol was 5.6 times lower and the concentration of carbamazepine was 3 times lower than levels in the surface water samples (Table 7).

**Table 7:** Pharmaceuticals in surface and drinking water in two First Nations (FN) located in the Mixedwood Plains in the Ontario region

Pharmaceutical detected	FN 1 surface water (ng/L)	FN 1 drinking water* (ng/L)	FN 2 surface water (ng/L)	FN 2 drinking water** (ng/L)
Acetaminophen	<10.0	<10.0	14	<10.0
Atenolol	38.8	6.9	42	<5.0
Bezafibrate	3.3	<1.0	7.8	<1.0
Caffeine	21.4	<5.0	502	96.2
Carbamazepine	28.4	9.2	45.7	<0.5
Cimetidine	3.8	<2.0	<2.0	<2.0
Ciprofloxacin	36	<20.0	29	<20.0
Clarithromycin	14.1	<2.0	35.3	<2.0
Clofibric acid	<1.0	<1.0	1.4	<1.0
Codeine	43.9	<5.0	101	<5.0
Cotinine	10.4	<5.0	50.7	14.4
Diclofenac	24	<15.0	38	<15.0
Diltiazem	5.2	<5.0	<5.0	<5.0
Diphenhydramine	<10.0	<10.0	14	<10.0
Furosemide	<5.0	<5.0	12.5	<5.0
Gemfibrozil	<1.0	<1.0	2.9	<1.0
Hydrochlorothiazide	55.2	<5.0	85.9	<5.0
Ibuprofen	<2.0	<2.0	85	<2.0
Metformin	404	<10.0	1550	<10.0
Metoprolol	25.6	<5.0	15.6	<5.0
Naproxen	<5.0	<5.0	120	<5.0
Ranitidine	33	<10.0	20	<10.0
Sulfamethazine	19.1	<5.0	10.9	<5.0
Sulfamethoxazole	45.7	<2.0	44.4	<2.0
Trimethoprim	6	<2.0	10.2	<2.0
Warfarin	<0.50	<0.50	1.76	<0.50
17α-Ethinylestradiol	0.74	<0.20	<0.20	<0.20

*In FN 1, five wells were sampled. Atenolol was detected at one site while carbamazepine was detected at another site

**In FN 2, five sites were sampled. Caffeine and cotinine were detected at three water sites

The second First Nation in Ontario, also located in the Mixedwood Plains ecozone, relied on groundwater wells for drinking water. In two water samples collected from five different wells, two pharmaceuticals were found: caffeine (a stimulant) and cotinine (a nicotine metabolite). The concentration of caffeine was 5.2 times lower, while that of cotinine was 3.5 times lower in the well water compared with the surface water (Table 7).

In the third First Nation, located in the Boreal Plains ecozone in Quebec, tap water samples were obtained from two households. One pharmaceutical, ketoprofen, was found in two tap water samples with a maximum level of 5.5 ng/L (Table 9) which is considered to be very low-level contamination. There are no health implications for ketoprofen at this level.

Finally, one First Nation, located in the Prairies ecozone in Alberta, requested the pharmaceutical sampling of their well; no pharmaceuticals were found.

Thus, among 11 drinking water sites sampled in four First Nations, five distinct pharmaceuticals were detected in three of them. Overall, 74 drinking water sources were sampled for this study (Table 2). However, only 11 drinking water samples were from treated water. As the treated water samples were at acceptable levels, no further sampling of treated drinking water samples was recommended.

### Comparison with drinking water guidelines

FNFNES results were compared with the Australian guidelines (2008), the California monitoring trigger levels (Anderson et al. 2010), and the New York State standards (NYCEP 2011) (Table 8).

**Table 8:** Comparison of FNFNES results in drinking water guidelines in Australia, California, and New York

Pharmaceutical	FNFNES max (ng/L) surface water	FNFNES max (ng/L) wastewater	FNFNES max (ng/L) drinking water	Australian guidelines	California monitoring trigger levels	New York State standard
Analgesic						
Codeine	101	563	0	50,000	NA	NA
Analgesic/anti-inflammatory						
Acetaminophen	307	14600	0	175,000	350,000	5000
Diclofenac	38	506	0	1800	1800	NA
Ibuprofen	367	15200	0	400,000	34,000	50,000
Ketoprofen	307	77.3	5.5	3500	3500	NA
Naproxen	244	4370	0	220,000	220,000	NA
Antacid						
Cimetidine	40.9	36.2	0	200,000	NA	NA
Ranitidine	33.0	238	0	NA	NA	NA
Antibiotic						
Chlortetracycline	12.0	0	0	105,000	NA	NA
Ciprofloxacin	37.7	7970	0	250,000	17,000	NA
Clarithromycin	69.6	929	0	250,000	NA	NA
Erythromycin	23.0	21	0	17,500	4900	NA
Isochlortetracycline	13	0	0	NA	NA	NA
Sulfamethazine	24.2	15.6	0	35,000	NA	NA
Sulfamethoxazole	87.0	2010	0	35,000	35,000	5000
Trimethoprim	32.0	696	0	70,000	61,000	NA
Anticoagulant						
Warfarin	6.9	171	0	NA	2300	NA
Anticonvulsant						
Carbamazepine	39.6	398	9.2	100,000	1000	50,000
Antidepressant						
Fluoxetine	50.7	0	0	10,000	10,000	NA
Antidiabetic						
Metformin	6210	17,700	0	250,000	NA	NA
Pentoxifylline	26.9	0	0	NA	NA	NA
Antihistamine						
Diphenhydramine	56	838	0	NA	NA	NA
Antihypertensive (beta-blocker)						
Atenolol	245	165	6.9	NA	70,000	NA
Metoprolol	77	26	0	25,000	25,000	NA
Antihypertensive						
Diltiazem	73.1	60.9	0	60,000	NA	5000
Diuretic						
Furosemide	30.7	128	0	NA	NA	NA
Hydrochlorothiazide	85.9	44.8	0	NA	NA	NA
Lipid regulator						
Bezafibrate	11.2	0	0	300,000	NA	NA
Clofibric acid	8.6	6.4	0	750,000	30,000	NA
Gemfibrozil	16.8	8.7	0	600,000	45,000	50,000
Nicotine metabolite (smoking cessation)						
Cotinine	80	1060	14.4	10,000	NA	50,000
Oral contraceptive						
17-α-Ethinylestradiol	0.74	0	0	1.5	280	NA
Statin						
Atorvastatin	8.8	5.6	0	5000	5000	NA
Stimulant						
Caffeine	4018	12,600	96.2	350	350	50,000

When comparing the FNFNES levels of pharmaceuticals found in source water (which is used by the community drinking water treatment plan), wastewater, and drinking water with the guidelines, the waters on First Nations would only be a concern for the levels of caffeine. As shown in Table 5, caffeine exceeded the Australian guideline and the California monitoring trigger level of 350 ng/L in First Nations situated in the Boreal Shield (Ontario Region), Hudson Plains (Ontario Region), Mixedwood Plains (Ontario Region), and Atlantic Maritime (Quebec Region) ecozones. However, the maximum level of caffeine found in this study is much lower than the New York standard of 50,000 ng/L.

### Pharmaceuticals in wastewater

In five First Nations (one located in the Hudson Plains ecozone in Ontario and four in the Prairies ecozone in Alberta and Saskatchewan) where five lagoons and one garbage dump pond were sampled, 28 unique pharmaceuticals were detected (Table 9). Concentrations were much higher than levels in the surface water samples from the same communities (Table 9). For example, analgesic/anti-inflammatory pharmaceuticals, such as acetaminophen and ibuprofen, were found in the lagoons at 14,600 and 15,000 ng/L, respectively. These values are more than 40 times higher than the values found in surface waters at 307 and 367 ng/L, respectively.

**Table 9:** Pharmaceuticals found in wastewater samples collected from five First Nations (one located in the Hudson Plains ecozone in Ontario and four in the Prairies ecozone in Alberta and Saskatchewan)

Pharmaceutical detected	FNFNES max (ng/L)	Quantitation limit (ng/L)	No. of samples analyzed	Range (ng/L)
Acetaminophen	14,600	10	8	15–14,600
Atenolol	165	5	7	17.9–165
Atorvastatin	5.6	5	1	5.6
Caffeine	12,600	5	10	91.2–12,600
Carbamazepine	398	0.5	10	0.53–398
Cimetidine	36.2	2	9	2.2–36.2
Ciprofloxacin	7970	20	5	58–7970
Clarithromycin	929	2	7	7.5–929
Clofibric acid	6.4	1	1	6.4
Codeine	563	5	9	7.4–563
Cotinine	1860	5	10	33.8–1860
Diclofenac	506	15	5	31–506
Diltiazem	60.9	5	2	59.3–60.9
Diphenhydramine	838	10	2	813–838
Erythromycin	21	10	2	18.0–21.0
Furosemide	128	5	2	121–128
Gemfibrozil	8.7	1	5	1.2–8.7
Hydrochlorothiazide	44.8	5	7	6.4–44.8
Ibuprofen	15,000	15	6	44–15,200
Ketoprofen	77.3	2	3	6.2–77.3
Metformin	17,700	10	10	223–17,700
Metoprolol	26.4	5	6	6.9–26.4
Naproxen	4370	5	10	13.1–4370
Ranitidine	238	10	5	22–238
Sulfamethazine	15.6	5	1	15.6
Sulfamethoxazole	2010	10	10	34.7–2010
Trimethoprim	696	2	8	14.5–696
Warfarin	171	0.5	3	1.31–171

The levels of pharmaceuticals in sewage treatment plants effluents were measured by several studies in Canada (Brun et al. 2006; Servos et al. 2005; Metcalfe et al. 2003; Saunders et al. 2016). In the four Atlantic regions, the max concentration of ibuprofen and naproxen was 35 ng/L whereas carbamazepine, an antiepileptic drug, was observed at the concentration of 79 ng/L (Brun et al., 2006). The mean concentration of 17β-estradiol in influent was 15.6 ng/L (range 2.4–26 ng/L) in conventional activated sludge and lagoon treatment systems and was reduced to 1.8 ng/L in final effluents (Servos et al. 2005).

### Human health risk from individual pharmaceuticals in water

The FNFNES results were compared with the guidelines established by Australia, California, and New York (Anderson et al. 2010; Australian Guidelines 2008; Drewes et al. 2018; NYCEP 2011). Only caffeine levels exceeded the Australian and California human health guidelines (Table 8). The concentrations of other individual pharmaceuticals detected in First Nations were unlikely to pose a threat to human health.

### Human health risk assessment of the mixture of pharmaceuticals

In this study, multiple pharmaceuticals were found in many surface water samples. Therefore, there was a concern that these contaminants in the waters may act synergistically. The evaluations of the exposure to pharmaceuticals undertaken by the WHO, Australia, California, and New York State determined the risk of exposure only to individual pharmaceuticals. However, in this study, mixtures of pharmaceuticals with up to 24 unique compounds were found in surface water and wastewater First Nations samples. Consequently, we evaluated the human health risk of exposure to pharmaceutical mixtures for all participating First Nations by using the Houtman method (Houtman et al. 2014). The results are presented only for four First Nations where multiple pharmaceuticals were detected in surface water, and therefore additional samples were taken (Tables 10, 11, 12, and 13).

**Table 10:** Human health risk assessment of pharmaceutical mixtures in one First Nation in the Boreal Shield ecozone in Ontario

Pharmaceutical	Max concentration (μg/L)	Health risk from mixture—females			% attribution
		ADI (μg/kg-d)	pGLV (μg/L)	BQ	
Metformin	5.64	7.1	21.3	0.264789	55.59
Cotinine	0.0442	0.28	0.84	0.052619	11.05
Atenolol	0.245	2	6	0.040833	8.57
Diphenhydramine	0.044	0.4	1.2	0.036667	7.70
Carbamazepine	0.0272	0.34	1.02	0.026667	5.60
Codeine	0.0711	1.4	4.2	0.016929	3.55
Gemfibrozil	0.0154	0.41	1.23	0.012520	2.63
Diltiazem	0.0597	1.7	5.1	0.011706	2.46
Clarithromycin	0.0693	7.1	21.3	0.003254	0.68
Naproxen	0.0611	6.3	18.9	0.003233	0.68
Sulfamethoxazole	0.087	10	30	0.002900	0.61
Ibuprofen	0.052	11.4	34.2	0.001521	0.32
Metoprolol	0.0611	14	42	0.001455	0.31
Trimethoprim	0.0302	20	60	0.000503	0.11
Bezafibrate	0.0112	8.6	25.8	0.000434	0.09
Hydrochlorothiazide	0.0056	6	18	0.000311	0.07
Caffeine	0.336	6667	20,001	0.000017	0.004
Pentoxifylline	0.0127	24,000	72,000	0.0000002	0.00004
Risk quotient				0.476356	

*ADI*, acceptable daily intake; *pGLV*, a provisional guideline value; *BQ*, benchmark quotient

**Table 11:** Human health risk assessment of pharmaceutical mixtures in the first of the two First Nations located in the Mixedwood Plains ecozone in Ontario (Ontario 1)

Pharmaceutical	Max concentration (μg/L)	Health risk from mixture—females			% attribution
		ADI (μg/kg-d)	pGLV (μg/L)	BQ	
17α-Ethinylestradiol	0.00074	0.000043	0.000129	5.4263570	98.47
Carbamazepine	0.0284	0.34	1.02	0.0278430	0.51
Metformin	0.325	7.1	21.3	0.0152580	0.28
Cotinine	0.0088	0.28	0.84	0.0104760	0.19
Codeine	0.0439	1.4	4.2	0.0104520	0.19
Ciprofloxacin	0.031	1.6	4.8	0.0064580	0.12
Atenolol	0.0375	2	6	0.0062500	0.11
Hydrochlorothiazide	0.0541	6	18	0.0030060	0.05
Sulfamethoxazole	0.0457	10	30	0.0015230	0.03
Ranitidine	0.033	11	33	0.0010000	0.018
Sulfamethazine	0.0191	10	30	0.0006370	0.012
Clarithromycin	0.0118	7.1	21.3	0.0005540	0.010
Metoprolol	0.0189	14	42	0.0004500	0.008
Cimetidine	0.0036	5.7	17.1	0.0002110	0.004
Bezafibrate	0.003	8.6	25.8	0.0001160	0.002
Trimethoprim	0.006	20	60	0.0001000	0.002
Caffeine	0.0214	6667	20,001	0.0000011	0.00002
Risk quotient				5.510692	

*ADI*, acceptable daily intake; *pGLV*, a provisional guideline value; *BQ*, benchmark quotient

**Table 12:** Human health risk assessment of pharmaceutical mixtures in the second of the two First Nations located in the Mixedwood Plains ecozone in Ontario (Ontario 2)

Pharmaceutical	Max concentration (μg/L)	Health risk from mixture—females			% attribution
		ADI (μg/kg-d)	pGLV (μg/L)	BQ	
Metformin	1.36	7.1	21.3	0.063850	33.53
Cotinine	0.0507	0.28	0.84	0.060357	31.69
Carbamazepine	0.0423	0.34	1.02	0.041471	21.78
Codeine	0.0339	1.4	4.2	0.008071	4.24
Atenolol	0.04	2	6	0.006667	3.50
Hydrochlorothiazide	0.0449	6	18	0.002494	1.31
Naproxen	0.0433	6.3	18.9	0.002291	1.20
Warfarin	0.00067	0.16	0.48	0.001396	0.73
Sulfamethoxazole	0.0391	10	30	0.001303	0.69
Gemfibrozil	0.0013	0.41	1.23	0.001057	0.56
Clarithromycin	0.0094	7.1	21.3	0.000441	0.23
Ranitidine	0.013	11	33	0.000394	0.21
Metoprolol	0.0124	14	42	0.000295	0.16
Bezafibrate	0.0029	8.6	25.8	0.000112	0.06
Acetaminophen	0.014	50	150	0.000093	0.05
Trimethoprim	0.0046	20	60	0.000077	0.04
Clofibric acid	0.0012	10	30	0.000040	0.021
Caffeine	0.115	6667	20,001	0.000006	0.003
Risk quotient				0.190416	

*ADI*, acceptable daily intake; *pGLV*, a provisional guideline value; *BQ*, benchmark quotient

**Table 13:** Human health risk assessment of pharmaceutical mixtures in wastewater site of a First Nation in the Prairies

Pharmaceutical	Max concentration (μg/L)	Health risk from mixture—females			% attribution
		ADI (μg/kg-d)	pGLV (μg/L)	BQ	
Cotinine	1.86	0.28	0.84	2.214286	36.9
Ciprofloxacin	7.97	1.6	4.8	1.660417	27.7
Metformin	17.4	7.1	21.3	0.816901	13.6
Diphenhydramine	0.838	0.4	1.2	0.698333	11.7
Naproxen	4.06	6.3	18.9	0.214815	3.58
Codeine	0.533	1.4	4.2	0.126905	2.12
Acetaminophen	14.6	50	150	0.097333	1.62
Sulfamethoxazole	2.01	10	30	0.067000	1.12
Diclofenac	0.031	0.5	1.5	0.020667	0.34
Furosemide	0.121	2.5	7.5	0.016133	0.27
Clarithromycin	0.278	7.1	21.3	0.013052	0.22
Diltiazem	0.0609	1.7	5.1	0.011941	0.20
Trimethoprim	0.682	20	60	0.011367	0.19
Carbamazepine	0.0108	0.34	1.02	0.010588	0.18
Ranitidine	0.219	11	33	0.006636	0.11
Hydrochlorothiazide	0.0357	6	18	0.001983	0.03
Cimetidine	0.0324	5.7	17.1	0.001895	0.03
Erythromycin	0.018	5	15	0.001200	0.02
Gemfibrozil	0.0012	0.41	1.23	0.000976	0.02
Metoprolol	0.0136	14	42	0.000324	0.01
Caffeine	1.15	6667	20,001	0.000058	0.001
Risk quotient				5.992809	

*ADI*, acceptable daily intake; *pGLV*, a provisional guideline value; *BQ*, benchmark quotient

Table 10 presents the human health risk from a mixture of 18 pharmaceuticals found in surface water in one First Nation located in the Boreal Shield ecozone in Ontario in the vicinity of a wastewater outflow. In this analysis, 55.6% of the risk can be attributed to metformin at 5640 ng/L whereas 11% of the risk was from cotinine at 44.2 ng/L. The RQ was at 0.47 (e.g., <1), so by this estimation, the risk was not a health concern.

Table 11 shows the calculation of the risk for the first of the two First Nations located in the Mixedwood Plains (Ontario 1). This First Nation is located downstream from a major urban centre in Ontario, where 17α-ethinylestradiol, at the level of 0.74 ng/L, was one of 17 distinct pharmaceuticals detected in a river under the influence of wastewater. The RQ was 5.5. Drinking this river water over a lifetime would pose a significant risk to human health (for example, affecting male and female reproduction, the thyroid and prostate functions), with 98.5 % of the risk coming from 17α-ethinylestradiol.

Table 12 shows the results for the second of the two First Nations located in the Mixedwood Plains in Ontario (Ontario 2). The concentrations of 18 pharmaceuticals detected in surface water were similar to those found in the first First Nation in Ontario (Table 11); however, 17α-ethinylestradiol was not found. In this First Nation, the risk to human health was low (RQ = 0.19) with over 91% of the risk coming from metformin (1360 ng/L and 33.5% of the risk), cotinine (50.7 ng/L and 31.7% of the risk), carbamazepine (42.3 ng/L and 21.8% of the risk), and codeine (33.9 ng/L and 4.2% of the risk). Consuming water from this river would not be considered a human health risk with respect to pharmaceutical concentrations.

For comparison, the RQ of 21 different pharmaceuticals found in the wastewater samples collected from a lagoon of a First Nation in the Alberta Prairies was 5.99 (Table 13), suggesting that the consumption of this water poses a human health hazard. In this site, 36.9% of the risk was attributed to cotinine at 1860 μg/L (with a potential increased risk of cardiovascular diseases) while 27.7% of the risk was from ciprofloxacin at 7970 μg/L (with an increased risk of liver disease), 13.6% of the risk was from metformin at 17,400 μg/L (with an increased risk of anemia), and 11.7% of the risk was from diphenhydramine at 838 μg/L (with a potential increased risk of nervous system disorders). Since the lagoon wastewater is not consumed, this risk assessment is for comparison purposes only.

These results showed that the risks of the mixtures of multiple pharmaceuticals detected in surface water in the First Nations were negligible. In community Ontario 1, however, drinking surface water over a lifetime would pose an elevated human health risk. Since the community members do not use surface water for drinking purposes, there should be no risks to human health.

## Conclusion

Overall, the source water of First Nations south of the 60^th^ parallel has low levels of pharmaceuticals and should not pose a threat to human health. However, in some locations, there were a variety of pharmaceuticals in surface water detected. Therefore, untreated surface water should not be used as an alternative water source. Also, five surface water sites in three First Nations were found to have a contraceptive, 17α-ethinylestradiol. Based on Houtman’s risk assessment of the mixture of pharmaceuticals, consuming those surface waters over a lifetime can pose potential risks to human health.

To reduce the presence of pharmaceuticals in the environment, in addition to reducing pharmaceutical dependence and over-prescriptions, it is recommended to develop a program that would assist First Nations in returning unused or expired prescription drugs, over-the-counter medications, and natural health products to a local pharmacy for proper disposal as an alternative to flushing them down the toilet or throwing them into the regular garbage. Future surface water monitoring is recommended as water sources and the level of water treatment vary by First Nation. Also, more comprehensive environmental studies that would examine the ecological effects of pharmaceuticals in the aquatic ecosystem are recommended.

## Supplementary information

ESM 1 (XLSX 30 kb)

ESM 2 (XLSX 20 kb)

## Footnotes

1 A total of 92 First Nations communities completed the five study components of FNFNES. Members from one First Nation community occupied territory in two ecozones so a decision was made to split the First Nation into two sites by an ecozone boundary. Therefore, this study describes a total of 93 First Nations at the AFN region and ecozone levels.
